# Ethanolic *Chromolaena odorata* (Siam weed) leaf extract exhibits broad-spectrum antimicrobial, antibiofilm, antioxidant, and cell-disruptive activities against clinically relevant bacteria

**DOI:** 10.14202/vetworld.2025.3982-3993

**Published:** 2025-12-18

**Authors:** Nattamol Phetburom, Thotsaporn Bunthiang, Siriwan Sunontarat, Peechanika Chopjitt, Rujirat Hatrongjit, Anusak Kerdsin, Suphachai Nuanualsuwan, Parichart Boueroy

**Affiliations:** 1 Department of Community Health, Faculty of Public Health, Kasetsart University, Chalermphrakiat Sakon Nakhon Province Campus, Sakon Nakhon 47000, Thailand; 2 Department of General Sciences, Faculty of Science and Engineering, Kasetsart University, Chalermphrakiat Sakon Nakhon Province Campus, Sakon Nakhon 47000, Thailand; 3 Department of Veterinary Public Health, Faculty of Veterinary Science, Chulalongkorn University, Bangkok, 10330, Thailand; 4 Center of Excellence for Food and Water Risk Analysis, Department of Veterinary Public Health, Faculty of Veterinary Science, Chulalongkorn University, Bangkok, 10330, Thailand

**Keywords:** antimicrobial activity, ethanolic *Chromolaena odorata* extract, biofilm inhibition, antioxidant activity, phytochemical profiling, *Bacillus cereus*, scanning electron microscopy, natural antibacterial agents

## Abstract

**Background and Aim:**

The rapid rise of antimicrobial resistance threatens effective infection control and reinforces the need for alternative therapeutics. *Chromolaena odorata* (Siam weed), a traditionally used medicinal plant rich in phenolic and flavonoid compounds, has been reported to possess antimicrobial properties. This study evaluated the antimicrobial, antibiofilm, antioxidant, and ultrastructural effects of ethanolic *C. odorata* leaf extract against a diverse panel of Gram-positive and Gram-negative bacteria.

**Materials and Methods:**

Ethanolic crude extract was prepared from dried *C. odorata* leaves, and its antimicrobial activity was assessed against 46 bacterial isolates using disk diffusion, minimum inhibitory concentration (MIC), and minimum bactericidal concentration (MBC) assays. Total phenolic and flavonoid contents were quantified using Folin–Ciocalteu and aluminum chloride methods. Antioxidant activity was measured using the 2,2-diphenyl picrylhydrazyl (DPPH) radical scavenging assay. Antibiofilm efficacy against *Bacillus cereus* was determined using crystal violet staining at sub-MIC levels. Ultrastructural alterations in *B. cereus* were examined via scanning electron microscopy (SEM).

**Results:**

The crude extract inhibited 78.26% (36/46) of tested isolates, with strong activity against nine species, including *B*. *cereus*, *Staphylococcus aureus*, *Staphylococcus epidermidis*, *Micrococcus luteus*, *Aeromonas hydrophila*, *Stenotrophomonas maltophilia*, *Citrobacter freundii*, and *Shigella sonnei*. MIC values ranged from 31.25–125 mg/mL, with *B. cereus* showing the lowest MIC and MBC (31.25 mg/mL). The extract exhibited high phenolic (96.82 ± 2.07 μg Gallat-equivalents/mg) and flavonoid (62.98 ± 2.64 μg Quercetin equivalent /mg) content, and moderate antioxidant activity (IC_50_ = 120.02 ± 16.31 μg/mL). Sub-MIC concentrations significantly inhibited *B. cereus* biofilm formation in a dose- and time-dependent manner, achieving up to 66.16% inhibition at 1/2 MIC after 72 h (p < 0.001). SEM analysis revealed cell shrinkage, wall collapse, and surface roughening in treated *B. cereus*, indicating disrupted cell integrity.

**Conclusion:**

Ethanolic *C. odorata* extract demonstrates broad-spectrum antibacterial, antibiofilm, antioxidant, and cell-disruptive activities, with pronounced effects against *B. cereus*. These findings highlight its potential as a natural antimicrobial or disinfectant candidate and support future development of plant-based agents to mitigate resistant bacterial infections.

## INTRODUCTION

Antibiotic resistance is an increasingly serious global challenge, with the emergence and spread of novel resistance mechanisms posing a significant threat to the effective treatment of common infectious diseases [1]. This growing resistance threatens to reverse the gains of modern medicine, as the safety and viability of critical medical procedures, including chemotherapy, organ transplantation, and routine surgery, such as cesarean section, are severely compromised when effective antimicrobial agents are unavailable [2]. Antibiotic-resistant bacteria contribute to increased medical costs, extended hospital stays, and higher mortality rates [3], and the failure of first-line antibiotics often necessitates the use of more costly therapeutic options.

*Chromolaena odorata* (Siam weed) is a toxic, globally widespread invasive species found across tropical and subtropical regions, including Thailand, West and Central Africa, Bangladesh, India, Laos, Sri Lanka, Cambodia, Taiwan, southern China, and Indonesia [4–6]. Despite its notoriety as an invasive weed, *C. odorata* contains rich phytochemicals, particularly flavonoids and tannins, which are known to interfere with bacterial adhesion, quorum-sensing, and biofilm matrix integrity. Its abundance and reported antimicrobial potential have made it an attractive candidate for exploring antibiofilm activity and ultrastructural effects on bacterial cells [7, 8].

The traditional use of *C. odorata* is widespread in many developing countries, where its fresh leaves and extracts are used to treat burns, skin infections, soft-tissue wounds, and postnatal wounds. It is also recognized for its antioxidant, anti-inflammatory, antimicrobial, antimalarial, cytoprotective, analgesic, and other therapeutic properties [9, 10]. Decoctions of *C. odorata* leaves are used as cough remedies, and mixtures with other plants, such as lemongrass and guava leaves, are traditionally used for malaria treatment [9]. Additional medicinal uses include its applications as an astringent, anti-diarrheal, anti-spasmodic, anti-hypertensive, tonic, diuretic, antipyretic, and cardiotonic agent [11]. Given these diverse bioactivities, identifying effective non-antibiotic natural compounds for preventing and treating bacterial infections is of considerable interest.

Previous studies have demonstrated that *C. odorata* leaf extracts possess antibacterial activity, with generally stronger effects against Gram-positive than against Gram-negative bacteria [7, 12, 13]. Methanolic and ethanolic extracts usually yield the highest antimicrobial potency, whereas aqueous extracts tend to be less active [13, 14]. Experimental evidence has reported inhibitory effects on *Bacillus cereus, Staphylococcus aureus, Enterococcus faecalis, Escherichia coli, Salmonella enterica serovar* Typhimurium, *Pseudomonas aeruginosa*, and *Streptococcus* [12–15]. Ethanolic extracts in particular exhibit antibiofilm activity against *P. aeruginosa* and *S. suis* and induce marked cellular damage [7, 15]. Isolated flavonoid compounds from *C. odorata* have further demonstrated potent antibacterial and antibiofilm activities, with minimum inhibitory concentrations ranging from 0.016 to 0.25 mg/mL [7]. The antibacterial components extracted by methanol or ethanol vary in activity and mechanism depending on the solvent, concentration, and target organism [16].

Despite substantial evidence of antibacterial potential, relatively few studies have assessed the specific antibiofilm effects of *C. odorata* or examined its morphological and ultrastructural impacts on bacterial cells. Most available research has focused on general antimicrobial action rather than biofilm disruption or cellular deformation [7, 17]. To address these gaps, the present study evaluated phytochemical composition, antimicrobial properties, minimum inhibitory concentration (MIC) and minimum bactericidal concentration (MBC) values, antioxidant capacity, antibiofilm activity, and ultrastructural alterations through a comprehensive experimental workflow. The findings may contribute valuable insights supporting the development of *C. odorata* extract as a natural antimicrobial agent for future applications.

Although *C. odorata* has been widely reported to possess antibacterial properties, most existing studies have focused primarily on its activity against planktonic bacteria, with limited exploration of its ability to inhibit biofilm formation, a critical factor in persistent and treatment-resistant infections. Furthermore, only a few investigations have examined how *C. odorata* affects bacterial cell morphology and ultrastructure, despite the importance of understanding cell-level responses to antimicrobial compounds. Previous work also varies greatly in extraction methods, concentrations, and target organisms, leading to inconsistent interpretations of potency and mechanism. The combined antimicrobial, antibiofilm, antioxidant, and cell-disruptive properties of ethanolic *C. odorata* extracts remain insufficiently characterized, particularly against clinically relevant Gram-positive and Gram-negative pathogens. These gaps highlight the need for a more integrative evaluation of its phytochemical profile and antibacterial mechanisms.

This study aimed to comprehensively evaluate the antimicrobial, antibiofilm, antioxidant, and ultrastructural effects of ethanolic *C. odorata* leaf extract against diverse Gram-positive and Gram-negative bacterial isolates. Specifically, the study sought to quantify its antimicrobial potency using disk diffusion, MIC, and MBC tests; determine its phytochemical composition and antioxidant capacity; assess its ability to inhibit biofilm formation at sub-MIC levels; and visualize structural alterations in bacterial cells using Scanning electron microscopy (SEM). By integrating these analyses, the study aimed to provide robust evidence supporting the potential application of *C. odorata* as a natural antimicrobial or disinfectant agent for controlling pathogenic bacteria, including those associated with antimicrobial resistance.

Although *C. odorata* has been reported to exhibit antimicrobial activity, most studies have focused primarily on planktonic bacterial growth, with limited investigation into its effects on biofilm formation and bacterial morphology. The workflow comprised extraction, phytochemical profiling, and antimicrobial assays, including MIC and MBC determinations, disk diffusion assays, biofilm inhibition testing, and an antioxidant assay. SEM was employed to assess bacterial cell integrity. The findings of this study may provide valuable information to support the development of *C. odorata* extract as a potential antimicrobial agent in future applications.

## MATERIALS AND METHODS

### Ethical approval

This study did not involve human participants or live animals. All experimental procedures, including plant extraction, microbial culture, and laboratory analyses, were conducted strictly in accordance with institutional biosafety and laboratory practice guidelines. Ethical approval was not required for this research.

### Study period and location

This study was conducted from March 2024 to March 2025. The extraction of *Chromolaena odorata* leaves was conducted at the Department of Thai Traditional Medicine, Faculty of Natural Resources, Rajamangala University of Technology Isan, Sakon Nakhon. The antimicrobial, antibiofilm, antioxidant, and SEM analysis was conducted at the Department of Community Health, Faculty of Public Health, Kasetsart University, Chalermprakiet Sakon Nakhon Campus, Sakon Nakhon Province, Thailand.

### Plant extraction

Samples of *C. odorata* leaves were collected from Sakhon Nakhon Province, Northeast Thailand (17.3014978 N, 104.1072305 E) [15]. *C. odorata* leaves were identified following the Thai plant names (Botanical names-vernacular names), 1980, by Assistant Prof. Ratchadawan Aukkanimart, Department of Thai Traditional Medicine, Faculty of Natural Resources, Rajamangala University of Technology Isan, Sakon Nakhon, Thailand. Voucher was deposited at the herbarium of the Department of Thai Traditional Medicine, and accession numbers were assigned as follows: leaves of *Chromolaena odorata* (L.) R.M.King & H.Rob., ASTERACEAE (Voucher No. Bouroey202401). The samples were washed thoroughly and dried in a hot-air oven at 60°C following established procedures [13]. The dried leaves were ground to a fine powder and weighed before extraction. A total of 250 g of powdered leaves was immersed in 2.5 L of 95% ethanol and macerated at room temperature for 7 days with occasional shaking. After maceration, the extract was filtered through Whatman No. 1 filter paper (GE Healthcare, UK). The filtrates were concentrated using an R-200 rotary evaporator (Büchi; Flawil, Switzerland) and stored in amber glass bottles at –20°C to protect them from oxidation and light degradation. The crude extract was dissolved in dimethyl sulfoxide (DMSO; Loba Chemie Pvt. Ltd., India) to prepare a 1 g/mL stock solution, which was kept at –20°C until further analysis. The percentage yield was calculated using:

Percentage yield = (Weight of dried extract / Weight of powdered leaves) × 100

### Bacterial strains and categorization

A total of 46 bacterial isolates, 15 Gram-positive and 31 Gram-negative strains, were tested (Table 1). These isolates were categorized based on their infection relevance into four groups: environmental or zoonotic pathogens (5 strains), enteric or foodborne pathogens (15 strains), skin and wound-associated pathogens (11 strains), and nosocomial or opportunistic pathogens (15 strains). Each isolate was maintained in 20% glycerol in trypticase soy broth at –20°C and subcultured on trypticase soy agar prior to experiments. All assays were conducted under strict aseptic conditions.

**Table 1:** Bacterial strains used in this study.

No.	Gram-positive	ATCC	No.	Gram-negative	ATCC
**Environmental or zoonotic pathogens**			26	*Staphylococcus epidermidis*	ATCC12228
1	*Edwardsiella tarda*	ATCC15947	27	*Staphylococcus epidermidis*	DMST15505
2	*Aeromonas hydrophila*	ATCC35654	28	*Staphylococcus haemolyticus*	ATCC15156
3	*Staphylococcus intermedius*	ATCC29663	29	*Staphylococcus saprophyticus*	DMST15906
4	*Acinetobacter lwoffii*	DMST19606	30	*Staphylococcus xylosus*	ATCC29971
5	*Acinetobacter lwoffii*	DMST25615	31	*Stenotrophomonas maltophilia*	ATCC13637
**Enteric or foodborne pathogens**			32	*Stenotrophomonas maltophilia*	DMST 25614
6	*Bacillus cereus*	ATCC11778	**Nosocomial or opportunistic pathogens**		
7	*Citrobacter freundii*	DMST16368	33	*Enterococcus faecalis*	AMR1545/20
8	*Citrobacter freundii*	ATCC43864	34	*Enterococcus faecalis*	AMR611/20
9	*Escherichia coli*	ATCC25922	35	*Enterococcus faecalis*	ATCC19433
10	*Klebsiella oxytoca*	ATCC49131	36	*Enterobacter hormaechei*	ATCC700323
11	*Morganella morganii*	ATCC25830	37	*Enterobacter cloacae*	A1238/20
12	*Proteus mirabilis*	ATCC35659	38	*Enterobacter cloacae*	ATCC3047
13	*Proteus vulgaris*	ATCC6380	39	*Klebsiella aerogenes*	ATCC51697
14	*Providencia stuartii*	ATCC33672	40	*Klebsiella pneumoniae*	ATCC-BAA1706
15	*Salmonella enterica*	ATCC13076	41	*Klebsiella pneumoniae*	ATCC35657
16	*Salmonella enterica*	ATCC13312	42	*Klebsiella pneumoniae*	DMST41335
17	*Salmonella enterica*	ATCC13314	43	*Klebsiella quasipneumoniae*	ATCC700603
18	*Shigella flexneri*	ATCC12022	44	*Klebsiella variicola*	ATCC-BAA 830
19	*Shigella sonnei*	ATCC25931	45	*Serratia marcescens*	ATCC13880
20	*Vibrio parahaemolyticus*	ATCC17802	46	*Pseudomonas aeruginosa*	ATCC27853
**Skin and wound-associated pathogens**					
21	*Bacillus subtilis*	ATCC6051			
22	*Micrococcus luteus*	ATCC4698			
23	*Staphylococcus aureus*	ATCC25923			
24	*Staphylococcus aureus*	A1471/20			
25	*Staphylococcus capitis*	ATCC35661			

ATCC = American Type Culture Collection

### Disk diffusion assay

The antimicrobial activity of *C. odorata* extract was assessed using the disk diffusion method following Clinical and Laboratory Standards Institute (CLSI, 2023) guidelines [18]. Bacterial suspensions were standardized to 0.5 McFarland (≈1 × 10^8^ CFU/mL) and inoculated onto Mueller-Hinton agar (MHA; HiMedia, India) in three directions using sterile swabs. Sterile 6-mm disks were loaded with 20 μL of extract at concentrations ranging from 25–500 mg/mL. Gentamicin (10 μg; Oxoid, UK) was used as the positive control, and DMSO as the negative control. Plates were incubated at 37°C for 18–24 h, and inhibition zones were measured. A minimum inhibition zone of ≥8 mm was used as the threshold for further testing [13].

### Determination of MIC and MBC

MIC and MBC values were determined using a two-fold serial dilution method in 96-well microplates with Mueller-Hinton broth (MHB; Difco™, France) [15]. Standardized inocula (0.5 McFarland ≈ 10^8^ CFU/mL) were further diluted to 10^6^ CFU/mL before adding 100 μL of bacterial suspension and 100 μL of extract at 25–500 mg/mL to each well. After 24 h incubation at 37°C, MIC was defined as the lowest extract concentration showing no visible turbidity. For MBC, 10 μL from MIC wells were plated on MHA and incubated for 24 h. The MBC was the lowest concentration, yielding no colony growth. All tests were performed in triplicate.

### Total phenolic content (TPC)

TPC was quantified using the Folin–Ciocalteu method [19]. Extracts (1 mg/mL) were mixed with 10% Folin–Ciocalteu reagent, distilled water, and 100 μL of 7.5% Na_2_CO_3_. After incubation in the dark for 30 min at room temperature, absorbance was recorded at 765 nm. TPC was calculated using a Gallic acid standard curve and expressed as mg Gallic acid equivalents (GAE) per gram of extract.

### Total flavonoid content (TFC)

TFC was determined using an aluminum chloride colorimetric method [19]. Extracts (1 mg/mL) were mixed with 10% AlCl_3_·6H_2_O and incubated in the dark for 15 min. Absorbance was measured at 415 nm, and flavonoid content was calculated using a quercetin standard curve, expressed as mg quercetin equivalents (QE) per gram of extract.

### Antioxidant activity (2,2-diphenyl picrylhydrazyl [DPPH] Assay)

The antioxidant capacity of extracts was assessed using the DPPH radical scavenging assay [19]. Extracts at concentrations of 31.25–500 μg/mL were mixed with a 0.4 mM DPPH solution and incubated in the dark for 30 min. Absorbance was measured at 517 nm. Antioxidant activity was expressed as IC_50_, defined as the concentration required to scavenge 50% of DPPH radicals, using Trolox as the reference standard.

### Biofilm inhibition assay

The antibiofilm activity of *C. odorata* extract was evaluated using a modified crystal violet assay [15]. Overnight cultures of *B. cereus* were diluted to 1 × 10^5^ CFU/mL in Tryptic Soy broth (Difco™), and 100 μL of bacterial suspension was mixed with 100 μL of extract at sub-MIC levels (1/2, 1/4, 1/8, and 1/16 MIC). Plates were incubated for 24, 48, and 72 h at 37°C. After incubation, wells were washed with phosphate-buffered saline (PBS; pH 7.4) three times, dried, stained with 0.1% crystal violet, washed again, and destained using 33% acetic acid. Absorbance was measured at 595 nm. Biofilm inhibition (%) was calculated as:

Biofilm inhibition (%) = [(OD control − OD treated) / OD control] × 100

### SEM

Ultrastructural changes in *B. cereus* were examined using SEM (JEOL, Japan). Bacterial cultures were treated with extract at MIC (31.25 mg/mL) and MIC/2 (15.625 mg/mL) and incubated overnight on glass coverslips. Cells were fixed in 2.5% glutaraldehyde, washed with PBS, dehydrated through graded ethanol (25%–100%), and sputter-coated with gold for 3 min at 25 mA. Samples were visualized at 10 kV with 7,500× magnification. Untreated cells served as controls.

### Statistical analysis

All experiments were performed in triplicate. Data are expressed as mean ± standard deviation. A one-way analysis of variance was used to analyze TPC, TFC, antioxidant activity, and antibiofilm assays. Significance levels were defined as *p < 0.05, **p < 0.01, and ***p < 0.001.

## RESULTS

### Antimicrobial activity screening using the disk diffusion method

**Figure 1:**
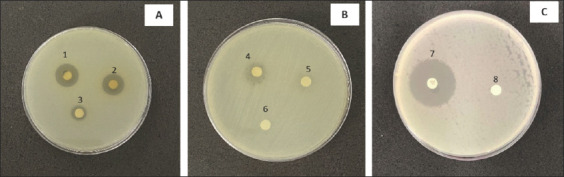
Inhibition zones of *Chromolaena odorata* crude extract against *Bacillus cereus* ATCC 11778. *B*. *cereus* ATCC 11778 was treated with crude extract at concentrations of (A) 125–500 mg/mL, (B) 25–100 mg/mL, and (C) control groups. 1 = 500 mg/mL, 2 = 250 mg/mL, 3 = 125 mg/mL, 4 = 100 mg/mL, 5 = 50 mg/mL, and 6 = 25 mg/mL, 7 = Gentamicin, 8 = control, ATCC = American Type Culture Collection.

**Figure 2:**
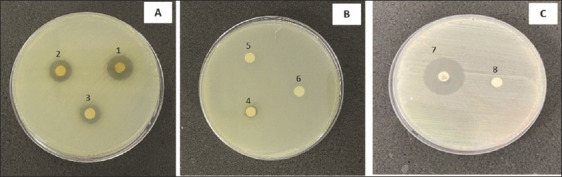
Inhibition zones of *Chromolaena odorata* crude extract against *Staphylococcus aureus* ATCC 25923. *S. aureus* ATCC 25923 was treated with crude extract at concentrations of (A) 125–500 mg/mL, (B) 25–100 mg/mL, and (C) control groups. Abbreviations: 1 = 500 mg/mL, 2 = 250 mg/mL, 3 = 125 mg/mL, 4 = 100 mg/mL, 5 = 50 mg/mL, and 6 = 25 mg/mL, 7 = Gentamicin, 8 = control, ATCC = American Type Culture Collection.

**Figure 3:**
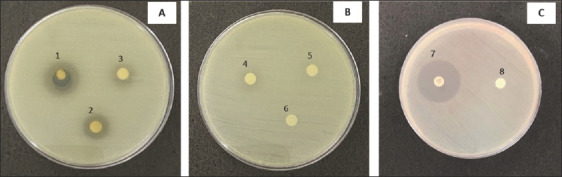
Inhibition zones of *Chromolaena odorata* crude extract against *Stenotrophomonas maltophilia* ATCC 13637. *S. maltophilia* ATCC 13637 was treated with crude extract at concentrations of (A) 125–500 mg/mL, (B) 25–100 mg/mL, and (C) control groups. Abbreviations: 1 = 500 mg/mL, 2 = 250 mg/mL, 3 = 125 mg/mL, 4 = 100 mg/mL, 5 = 50 mg/mL, and 6 = 25 mg/mL, 7 = Gentamicin, 8 = control, ATCC = American Type Culture Collection.

The percentage yield of the crude *C. odorata* leaf extract was 5%. The extract inhibited 78.26% (36/46) of the tested bacterial isolates, comprising 50% Gram-negative (23/46) and 28.26% Gram-positive (13/46) bacteria (Table 2). Strong inhibitory activity was observed against nine species: *B. cereus*, *S. epidermidis*, *Micrococcus luteus*, *S. aureus*, *Vibrio parahaemolyticus*, *Aeromonas hydrophila*, *Shigella sonnei*, *Stenotrophomonas maltophilia*, and *Citrobacter freundii*, with inhibition zones ranging from 12–18, 9–14, 12–11, 8–16, 7–10, 9–10, 7–12, 7–13, and 7–10 mm, respectively. These findings indicate broad inhibitory potential against both Gram-positive bacteria (e.g., *B. cereus*; Figure 1 and *S. aureus*; Figure 2) and Gram-negative bacteria (Figure 3). Overall, the *C. odorata* extract demonstrated stronger activity against Gram-negative isolates than Gram-positive isolates.

**Table 2:** Antimicrobial activity (zone of inhibition, mm, and MIC and MBC values, mg/mL) of *Chromolaena odorata* leaves extract against both Gram-positive and Gram-negative bacteria.

A	B	C	D	E	F	G	H	I	J	K
Gram-positive	*Bacillus cereus* ATCC 11778	18 ± 0.57	16 ± 1.00	13 ± 0.57	12 ± 0.28	ND	ND	26 ± 1	31.25	31.25
	*Staphylococcus epidermidis* ATCC 12228	14 ± 0.72	12 ± 0.25	10 ± 1.00	9 ± 0.28	ND	ND	24 ± 1	31.25	125
	*Micrococcus luteus* ATCC 4698	12 ± 1.25	11 ± 0.68	11 ± 0.96	ND	ND	ND	16 ± 1	31.25	125
	*Staphylococcus aureus* ATCC 25923	16 ± 0.76	12 ± 0.28	9 ± 0.58	8 ± 0.28	ND	ND	27 ± 1	62.5	>250
	*Vibrio parahaemolyticus* ATCC 17802	10 ± 0.60	9 ± 0.90	8 ± 0.79	7 ± 0.76	ND	ND	16 ± 1	31.25	62.5
Gram-negative	*Aeromonas hydrophila* ATCC 35654	10 ± 0.05	9 ± 0.46	9 ± 0.23	9 ± 0.26	ND	ND	15 ± 1	62.5	250
	*Shigella sonnei* ATCC 25931	12 ± 0.50	7 ± 0.45	ND	ND	ND	ND	15 ± 1	62.5	250
	*Stenotrophomonas maltophilia* ATCC 13637	13 ± 0.76	9 ± 0.55	8 ± 0.77	7 ± 0.10	ND	ND	22 ± 1	125	>250
	*Citrobacter freundii* ATCC 43864	10 ± 0.43	7 ± 0.05	ND	ND	ND	ND	21 ± 1	31.25	250

A = Gram stain, B = Microorganism, C = Diameter of zone of inhibition (mm) at 500 mg/mL, D = Diameter of zone of inhibition (mm) at 250 mg/mL, E = Diameter of zone of inhibition (mm) at 125 mg/mL, F = Diameter of zone of inhibition (mm) at 100 mg/mL, G = Diameter of zone of inhibition (mm) at 50 mg/mL, H = Diameter of zone of inhibition (mm) at 25 mg/mL, I = Diameter of zone of inhibition (mm) for gentamicin (GM), J = Minimum inhibitory concentration (MIC, mg/mL), K = Minimum bactericidal concentration (MBC, mg/mL), MIC = Minimum inhibitory concentration, MBC = Minimum bactericidal concentration, ATCC = American Type Culture Collection, GM = Gentamicin, ND = No activity, *B. cereus* = *Bacillus cereus*, *S. epidermidis = Staphylococcus epidermidis, M. luteus = Micrococus luteus, S. aureus = Staphylococcus aureus, V. parahaemolyticus = Vibrio parahaemolyticus, A. hydrophila = Aeromonas hydrophila, S. sonnei = Shigella sonnei, S. maltophilia = Stenotrophomonas maltophilia, C. freundii = Citrobacter freundii*

### MIC and MBC

The crude extract showed the highest antimicrobial activity against *B. cereus*, *S. aureus*, *V. parahaemolyticus*, *M. luteus*, and *S. sonnei*, with MICs of 31.25 mg/mL for each (Table 2). Higher MIC values were observed for *S. maltophilia*, *A. hydrophila*, and *S. epidermidis* (62.5 mg/mL) and *C. freundii* (125 mg/mL). The MBC value was 31.25 mg/mL for *B. cereus*, while MBC values for *V. parahaemolyticus*, *S. aureus*, *M. luteus*, and *S. sonnei* were 62.5, 125, 125, and 125 mg/mL, respectively. MBC values for *S. maltophilia* and *A. hydrophila* were 250 mg/mL, and those for *S. epidermidis* and *C. freundii* exceeded 250 mg/mL (Table 2).

### Phenolic and flavonoid contents and antioxidant properties

The TPC of the ethanolic extracts was 96.82 ± 2.07 mg GAE/g, while the TFC was 62.98 ± 2.64 mg QE/g of extract (Table 3). The extract exhibited DPPH radical scavenging activity, with an IC_50_ value of 120.02 ± 16.31 μg/mL (Table 4). Although antioxidant activity was evident, it was significantly lower than that of the Trolox positive control (p < 0.01).

**Table 3:** Total phenolic and total flavonoid constituents of ethanolic *Chromolaena odorata* extract.

Sample	TPC (mgGA/g extracts)	TFC (mgQE/g extracts)
Ethanolic *C. odorata* extract	96.82 ± 2.07	62.98 ± 2.64

TPC = Total phenolic content, TFC = Total flavonoid content

**Table 4:** DPPH radical scavenging activities expressed as 50% inhibitory concentration (IC_50_) of ethanolic *Chromolaena odorata* extract.

Sample	2,2-diphenyl picrylhydrazyl (IC_50_) (μg/mL)
Ethanolic *C. odorata* extract	120.02 ± 16.31**
Trolox	25.88 ± 1.06

### Inhibition of bacterial biofilm formation

**Figure 4:**
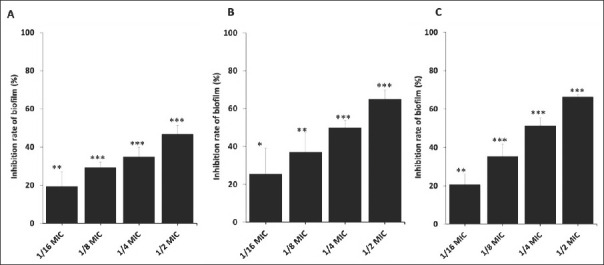
Biofilm formation by *Bacillus cereus* in the presence of *Chromolaena odorata* crude leaf extract at sub-inhibitory concentrations. Quantification of crystal violet dye attached to *B. cereus* cells forming biofilms after treatment with crude extract with 1/2 MIC, 1/4 MIC, 1/8 MIC, and 1/16 MIC at (A) 24, (B) 48, and (C) 72 h. *p < 0.05, **p < 0.01, ***p < 0.001. MIC = Minimum inhibitory concentration.

*C. odorata* extract significantly reduced *B. cereus* biofilm formation in both a concentration- and time-dependent manner (Figure 4). At 24 h, biofilm inhibition rates were 46.82% (1/2 MIC; p < 0.001), 34.84% (1/4 MIC; p < 0.001), 29.25% (1/8 MIC; p < 0.001), and 19.51% (1/16 MIC; p < 0.01) (Figure 4A). At 48 h, inhibition increased to 64.96% (1/2 MIC; p < 0.001), 49.91% (1/4 MIC; p < 0.001), 36.99% (1/8 MIC; p < 0.01), and 25.47% (1/16 MIC; p < 0.05) (Figure 4B). At 72 h, the inhibition rates were 66.16% (1/2 MIC; p < 0.001), 51.30% (1/4 MIC; p < 0.001), 35.41% (1/8 MIC; p < 0.001), and 20.66% (1/16 MIC; p < 0.01) (Figure 4C). Across all time points, the extract maintained progressive and dose-dependent antibiofilm efficacy.

### SEM observations

**Figure 5:**
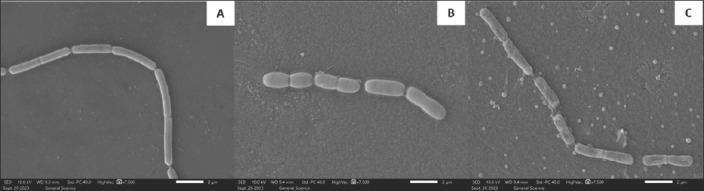
Scanning electron microscopic (SEM) images of *Bacillus cereus* on a glass slide after incubation with different concentrations of *Chromolaena odorata* leaves crude extract. SEM images of *B. cereus* were obtained after treatment with (A) 0 mg/mL, (B) 15.625 mg/mL (1/2 MIC), and (C) 31.25 mg/mL (MIC). Magnification 7,500× at 10.0 kV. The bar at the bottom right means 2 μm. MIC = Minimum inhibitory concentration.

SEM analysis revealed marked morphological alterations in *B. cereus* following exposure to the extract (Figure 5). Cells in the untreated control and those exposed to MIC/2 exhibited intact, smooth surfaces with well-defined cellular structures (Figures 5A and 5B). In contrast, cells treated with the MIC showed substantial surface roughening, shrinkage, deformation, and signs of cell wall destruction (Figure 5C). These structural abnormalities strongly indicate compromised cell integrity and loss of viability.

## DISCUSSION

### Comparison of extraction yields with previous studies

Previous studies [13, 20, 21] have reported extraction yields of *C. odorata* leaf extracts ranging from 8.42%–10.45% using ethanol as the solvent. In contrast, the present study obtained an extraction yield of approximately 5%. Despite this lower yield, previous research consistently shows that aqueous, ethanolic, and methanolic extracts of *C. odorata* possess notable antimicrobial activity against a wide range of Gram-positive and Gram-negative pathogens [11, 22–26]. Our earlier work similarly demonstrated 100% inhibition of *S. suis* strains, with MIC and MBC values ranging from 3.9 to 62.5 mg/mL [15].

### Comparative antimicrobial efficacy across pathogens

The antimicrobial potency observed in this study aligns with several earlier investigations. Crude leaf extracts have shown antimicrobial activity ranging from 0.156 to 1.25 mg/mL against *K. oxytoca*, *S. sonnei*, *S. enterica*, and *V. cholerae* [27]. Similarly, ethanolic extracts exhibited inhibitory effects against *E. coli* DMST 4212 (MIC: 1.25 mg/mL; MBC: 2.50–5.00 mg/mL) [28]. A previous study by Alabi *et al*. [29] has reported antimicrobial activity against *Providencia vermicola*, *Proteus mirabilis*, and *P. aeruginosa*, with MIC and MBC ranges of 12.5–25 mg/mL and 100–200 mg/mL, respectively. The MIC and MBC ranges obtained in this study (31.25–62.5 mg/mL and 31.25 to >250 mg/mL) further confirm the broad-spectrum potential of *C. odorata* extracts against clinically important bacteria.

### Structural differences in Gram-positive and Gram-negative bacteria

Differences in susceptibility between bacterial groups can be attributed to structural variations in their cell envelopes. Gram-negative bacteria possess an outer phospholipid membrane rich in lipopolysaccharide, which restricts the penetration of lipophilic compounds [9]. Conversely, Gram-positive bacteria have a thick peptidoglycan layer, which does not serve as an effective permeability barrier [11]. The antimicrobial flavonoids found in *C. odorata* are known to bind the bacterial cell wall, disrupt biosynthesis, and inhibit growth [30, 31], a mechanism consistent with the morphological alterations observed in this study.

### Ultrastructural alterations and cell damage mechanisms

SEM analysis revealed substantial morphological changes in *B. cereus* following treatment, including cell shrinkage, surface roughness, and structural collapse, findings consistent with previous studies. Extracts of *Ganoderma lucidum* and *G. neo-japonicum* induced similar shrinkage and lysis in *S*. Typhimurium, *Salmonella* Enteritidis, and *Escherichia coli* [32], while *Centella asiatica* extract caused roughening, wrinkling, and membrane rupture in *V. alginolyticus* [33]. Our previous work also reported that *C. odorata* extracts compromised *S. suis* cell integrity, causing surface wrinkling, leakage of intracellular contents, and cell lysis [15]. Likewise, green tea polysaccharides resulted in roughened membranes and cytoplasmic leakage in *E. coli* DH5α [34]. These collective findings reinforce the notion that *C. odorata* possesses strong cell-disruptive properties, mediated by interactions with cellular envelopes.

### Role of biofilm resistance and anti-biofilm effects

Biofilms, composed of structured microbial communities embedded in extracellular polymeric substances, are notably resistant to antimicrobial agents and host immune responses, contributing to poor clinical outcomes [35]. In this study, *C. odorata* extract significantly inhibited *B. cereus* biofilm formation in a dose- and time-dependent manner. This aligns with previous findings where *C. odorata* extract suppressed biofilm formation in *S. suis* and *P. aeruginosa* [15, 36]. Comparable antibiofilm activity has been reported for ethanolic *Piper betle* leaf extract against *S. aureus* and *E. coli* [37] and methanolic *Verbena tenuisecta* leaf extract against *E. coli* [38].

### Phytochemical constituents and their biological actions

*C. odorata* contains diverse bioactive constituents, predominantly flavonoids and tannins, which exhibit antimicrobial, antioxidant, and antibiofilm activities [39]. Tannins induce bacterial cell wall or membrane shrinkage, disrupt permeability, and inhibit cell growth or cause cell death [39, 40]. They also interfere with microbial adhesion, enzyme activity, and transport proteins [39]. Secondary metabolites, such as alkaloids, flavonoids, and tannins, are recognized contributors to antimicrobial activity [41]. In this study, the high total phenolic and flavonoid contents corroborate previous findings [42] and help explain the extract’s strong antioxidant and antimicrobial actions. These phytochemicals not only directly scavenge free radicals [19] but also enhance antibacterial efficiency.

### Mechanisms underlying antibiofilm activity

Research suggests that the antibiofilm properties of *C. odorata* are largely attributable to its flavonoid and tannin content [7, 8]. Flavonoids disrupt biofilms by interfering with quorum-sensing pathways, inhibiting bacterial adhesion, and suppressing the synthesis of extracellular polymeric substances. Ultrastructural analyses show that flavonoids disrupt membrane integrity by intercalating into phospholipid layers, leading to thinning and fragmentation of the biofilm matrix. Tannins further destabilize biofilms by precipitating cell-surface proteins, inhibiting adhesins, and chelating essential ions, thereby compromising enzymatic functions and structural stability [17]. Together, these mechanisms explain the pronounced reduction in biofilm mass and the cellular deformation observed in this study. Given the global rise of antimicrobial resistance, natural compounds that act on both planktonic and biofilm forms of pathogens contribute significantly to the WHO’s One Health framework for sustainable infection control [43].

### Proposed antimicrobial mechanism and future applications

**Figure 6:**
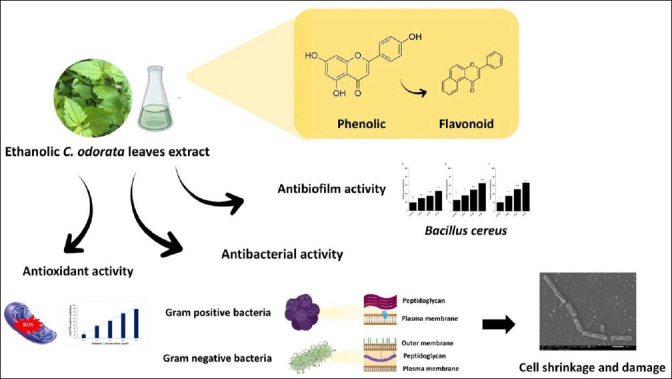
Schematic representation of the properties of ethanolic *Chromolaena odorata* extract.

The proposed antimicrobial mechanism of *C. odorata* crude extract involves disruption of cell wall integrity, alteration of membrane permeability, and interference with biosynthetic pathways. Flavonoids may bind to bacterial cell walls, inhibit peptidoglycan synthesis, and lead to cell death [31]. A schematic summary of these mechanisms is presented in Figure 6. Based on the antimicrobial and antibiofilm activities observed, *C. odorata* extract shows promise for development as a natural antimicrobial agent or surface decontaminant.

## CONCLUSION

This study demonstrates that ethanolic *C. odorata* leaf extract possesses substantial antibacterial, antibiofilm, antioxidant, and cell-disruptive properties against a wide range of clinically relevant Gram-positive and Gram-negative bacteria. The extract exhibited a 5% yield and inhibited 78.26% (36/46) of all tested isolates, with particularly strong activity against *B. cereus*, *S. aureus*, *S. epidermidis*, *M. luteus*, *V. parahaemolyticus*, *A. hydrophila*, *S. sonnei*, *S. maltophilia*, and *C. freundii*. MIC and MBC values ranged from 31.25 to >250 mg/mL. High levels of total phenolics (96.82 ± 2.07 mg GAE/g) and flavonoids (62.98 ± 2.64 mg QE/g) corresponded with the extract’s moderate antioxidant capacity (IC_50_ = 120.02 ± 16.31 μg/mL). The extract also significantly inhibited *B. cereus* biofilm formation, up to 66.16% at 1/2 MIC after 72 h, and SEM imaging confirmed marked ultrastructural destruction, including membrane collapse, shrinkage, and surface roughening.

From a practical perspective, these findings suggest that *C. odorata* has promising potential as a natural antimicrobial alternative or adjunct, particularly in settings where antibiotic resistance restricts treatment options. Its efficacy against both planktonic and biofilm-associated bacteria underscores its relevance for food safety, wound care, and the disinfection of contaminated surfaces. The plant’s wide availability and established traditional use further support its feasibility for low-cost antimicrobial development.

The main strengths of this study include its comprehensive experimental design which combines phytochemical analysis, antimicrobial profiling, antibiofilm assessment, and SEM evaluation, enabling a detailed mechanistic understanding of the extract’s activity. Using multiple species of Gram-positive and Gram-negative pathogens also improves the generalizability of the findings.

However, the study has limitations. All experiments were performed *in vitro*, and therefore the biological activity of *C. odorata* extract under in vivo conditions, including toxicity, pharmacokinetics, stability, and tissue compatibility, remains unknown. The crude extract composition was not fractionated, and specific bioactive constituents responsible for the observed effects were not isolated. Additionally, synergistic or antagonistic interactions with conventional antibiotics were not evaluated.

Future research should focus on isolating active compounds, characterizing their molecular mechanisms, and assessing their safety profiles using animal models. Synergy studies with clinically used antibiotics may reveal combination strategies that enhance antimicrobial efficacy while reducing resistance pressure. Evaluating the extract’s potential as a topical disinfectant, food preservative, or biofilm-control agent may also broaden its applied relevance. A One Health–aligned approach integrating environmental, veterinary, and human-health perspectives will help position *C. odorata* as a sustainable natural antimicrobial resource.

In conclusion, ethanolic *C. odorata* leaf extract exhibits multi-targeted antimicrobial, antibiofilm, and structural-disruptive activities supported by its rich phytochemical composition. Although additional work is required to validate its therapeutic safety and clinical relevance, the extract represents a promising natural candidate for combating persistent and resistant bacterial pathogens and offers valuable potential for future development of plant-based antimicrobial solutions.

## DATA AVAILABILITY

The supplementary data can be made available from the corresponding author upon request.

## AUTHORS’ CONTRIBUTIONS

NP: Performed the laboratory examination, data collection, and drafting of the manuscript. TB and SS: Performed the laboratory examination and data collection. PC and RH: Analyzed the data and data collection. A K and SN: Analyzed and interpreted the data. PB: Contributed to the study design, data interpretation, and conceptual framework, and edited the manuscript. All authors have reviewed and approved the final version of the manuscript.

## References

[1] Tang Q, Song P, Li J, Kong F, Sun L, Xu L (2016). Control of antibiotic resistance in China must not be delayed:the current state of resistance and policy suggestions for the government, medical facilities, and patients. Biosci. Trends.

[2] Uddin T. M, Chakraborty A. J, Khusro A, Zidan B.R.M, Mitra S, Emran T. B, Dhama K, Ripon M.K.H, Gajdács M, Sahibzada M.U.K, Hossain M. J (2021). Antibiotic resistance in microbes:History, mechanisms, therapeutic strategies and future prospects. J. Infect. Public Health.

[3] Imarenezor E.P.K, Ebuara F. U, Abhadionmhen O. A, Brown S.T.C, Isaac K (2020). Antimicrobial effects of *Chromolaena odorata* leaves on *Staphylococcus aureus* and *Streptococcus* spp. isolates from urine of patients attending general hospital, Wukari, North East Nigeria. GSJ.

[4] Mapook A, Hyde K. D, McKenzie E. H, Jones E. G, Bhat D. J, Jeewon R, Stadler M, Samarakoon M. C, Malaithong M, Tanunchai B, Buscot F (2020). Taxonomic and phylogenetic contributions to fungi associated with the invasive weed *Chromolaena odorata* (Siam weed). Fungal Divers.

[5] Hung T. M, Cuong T. D, Dang N. H, Zhu S, Long P. Q, Komatsu K, Min B. S (2011). Flavonoid glycosides from *Chromolaena odorata* leaves and their in vitro cytotoxic activity. Chem. Pharm. Bull.

[6] Umukoro S, Ashorobi R. B (2006). Evaluation of the anti-inflammatory and membrane stabilizing effects of *Eupatorium odoratum*. Int. J. Pharma.

[7] Omokhua-Uyi A. G, Abdalla M. A, Leonard C. M, Aro A, Uyi O. O, Van Staden J, McGaw L. J (2020). Flavonoids isolated from the South African weed *Chromolaena odorata* (Asteraceae) have pharmacological activity against uropathogens. BMC Complement. Med. Ther.

[8] do Nascimento J. B, da Costa J. G. M (2025). Flavonoids:A review of antibacterial activity against Gram-negative bacteria. Int. J. Microbiol.

[9] Ayyanar M, Ignacimuthu S (2009). Herbal medicines for wound healing among tribal people in Southern India:Ethnobotanical and scientific evidences. Int. J. Appl. Res. in Nat. Prod.

[10] Pragadheeswari R, Sangeetha K (2016). Herbal finishing for anti-bacterial property with *Chromolaena odorata* herb. Int. J. Home Sci.

[11] Vital P. G, Windell L. R (2011). Antimicrobial activity and cytotoxicity of *Chromolaena odorata* (L. f.) King and Robinson and *Uncaria perrottetii* (A. Rich) Merr. Extracts. J. Med. Plants Res.

[12] Odutayo F, Ezeamagu C, Kabiawu T, Aina D, Mensah-Agyei G (2017). Phytochemical screening and antimicrobial activity of Chromolaena odorata leaf extract against selected microorganisms. J. Adv. Med. Pharm. Sci.

[13] Thophon S.H.S, Waranusantigul P, Kangwanrangsan N, Krajangsang S (2016). Antimicrobial activity of Chromolaena odorata extracts against bacterial human skin infections. Mod. Appl. Sci.

[14] Indrianingsih A. W, Ahla M. F, Windarsih A, Wiyono T, Noviana E, Fadzhillah N. A, Alfiani R. N (2025). Phytochemical constituent of devil weed (Chromolaena odorata), concurrent with its antioxidant, α-glucosidase inhibitory, and antibacterial activity. Molecules.

[15] Phetburom N, Chopjitt P, Dulyasucharit R, Nontunha N, Daenprakhom K, Ongarj P, Hatachote S, Srichaijaroonpong S, Hatrongjit R, Kerdsin A, Boueroy P (2025). Antimicrobial activity of Chromolaena odorata crude extracts against Streptococcus suis. Microb. Pathog.

[16] Nanadini N, Nagababu P, Rao V. U, Venugopal N (2014). Phytochemical, antimicrobial and antioxidant properties of an invasive weed Chromolaena odorata (L.) King &Robinson. Int. J. Phytomed.

[17] Villanueva X, Zhen L, Ares J. N, Vackier T, Lange H, Crestini C, Steenackers H. P (2023). Effect of chemical modifications of tannins on their antimicrobial and antibiofilm effect against Gram-negative and Gram-positive bacteria. Front. Microbiol.

[18] Clinical and Laboratory Standards Institute (CLSI) (2023). Performance Standards for Antimicrobial Susceptibility Testing, 33rd ed.

[19] Promchiang J, Aukkanimart R, Sriraj P (2025). Anti-hepatocellular carcinoma and antioxidant activities of a Thai traditional liver disease formulation:GC–MS and FTIR profiling. J. Appl. Pharm. Sci.

[20] Prastiyanto M. E, Ni Made B.A.D, Pratiningtias T. D, Ni Made R. P, Windayani A, Wahyunengsih E, Amir E, Wardoyo F. A (2021). In vitro antibacterial activities of crude extracts of nine plants on multidrug resistance bacterial isolates of wound infections. Biodiversitas.

[21] Oko J. O, Audu J. A, Ojeleye F. S, Okey Q, Jakheng S. P, Shittu K. J (2017). Comparative assessment of antibacterial activity of Chromolaena odorata leaf extracts against selected clinical bacterial isolates. J. Adv. Microbiol.

[22] Naidoo K. K, Coopoosamy R. M, Naidoo G (2011). Screening of Chromolaena odorata (L.) King and Robinson for antibacterial and antifungal properties. J. Med. Plant Res.

[23] Ojha D, Maity C, Mohapatra P. D, Samanta A (2010). In vitro antimicrobial potentialities of different solvent extracts of ethnomedicinal plants against clinically isolated human pathogens. J. Phytol.

[24] Mondal K. C, Bhargava D, Shivapuri J. N, Kar S (2012). In vitro antigonorrhoeal activity and extraction of chemical constituents from the leaves of Chromolaena odorata (Lin.) locally known as 'BANMARA'. Int. J. Chem. Anal. Sci.

[25] Stanley M. C, Ifeanyi O. E, Nwakaego C. C, Esther I. O (2014). Antimicrobial effects of Chromolaena odorata on some human pathogens. Int. J. Curr. Microbiol. Appl. Sci.

[26] Eze E. A, Oruche N. E, Onuora V. C, Eze C. N (2013). Antibacterial screening of crude ethanolic leaf extracts of four medicinal plants. J. Asian Sci. Res.

[27] Atindehou M, Lagnika L, Guérold B, Strub J. M, Zhao M, Van Dorsselaer A, Marchioni E, Prévost G, Haikel Y, Taddéi C, Sanni A (2013). Isolation and identification of two antibacterial agents from Chromolaena odorata L. active against four diarrheal strains. Adv. Microbiol.

[28] Bunkaew N, Saensena P, Boonpeng P, Sumranmak P, Hanpipatsatian T, Panutai W (2025). Antioxidant and antibacterial potential of Chromolaena odorata (L.) medicinal plant extracts. J. Biol. Res.

[29] Alabi M, Olusola-Makinde O, Oladunmoye M. K (2024). Unlocking the antibacterial efficacy of Chromolaena odorata extract in wound healing. Microbes Infect. Dis.

[30] Lavanya G, Brahmaprakash G. P (2011). Phytochemical screening and antimicrobial activity of compounds from selected medicinal and aromatic plants. IJSN.

[31] Anyasor G. N, Aina D. A, Olushola M, Aniyikaye A. F (2011). Phytochemical constituent, proximate analysis, antioxidant, antibacterial and wound healing properties of leaf extracts of Chromolaena odorata. Ann. Biol. Res.

[32] Ayimbila F, Siriwong S, Chaiyama V, Srihanant N, Keawsompong S (2023). Comparative study of bio-functional profile and bioactivities of polysaccharides from Ganoderma lucidum and Ganoderma neo-japonicum. Biocatal. Agric. Biotechnol.

[33] Ibrahim W.N.W, Huzmi H, Aznan A. S, Iberahim N. A, Zamani A. I, Laith A, Kamal A.H.M, Sheikh H. I, Nadirah M, Wan-Zaliha W. S, Najiah M (2023). *In vitro* and *in vivo* characterisations of *Centella asiatica* extract against *Vibrio alginolyticus* infection in whiteleg shrimp, *Penaeus vannamei*. Songklanakarin J. Sci. Technol.

[34] Zhou Y, Yao Q, Zhang T, Chen X, Wu Z, Zhang N, Shao Y, Cheng Y (2020). Antibacterial activity and mechanism of green tea polysaccharide conjugates against *Escherichia coli*. Ind. Crops Prod.

[35] Azeem K, Fatima S, Ali A, Ubaid A, Husain F. M, Abid M (2025). Biochemistry of bacterial biofilm:insights into antibiotic resistance mechanisms and therapeutic intervention. Life.

[36] Wan Mohd Zawawi W.M.A, Ibrahim M.S.A, Rahmad N, Hamid U.M.A, Raja Yahya M.F.Z (2020). Proteomic analysis of *Pseudomonas aeruginosa* biofilm treated with *Chromolaena odorata* extracts. Malays. J. Microbiol.

[37] Saeloh D, Visutthi M (2021). Efficacy of Thai plant extracts for antibacterial and anti-biofilm activities against pathogenic bacteria. Antibiotics.

[38] Azeem A, Rahman A. U, Iqbal A, Kausar F, Gul S, Khalil A.A.K, Hakeem K. R, Noor S, Zari A, Zaman M, Alghamdi K. M (2025). Phytochemical Composition and Bioactivities of *Verbena tenuisecta* (Briq.) Leaf Extracts:Antimicrobial and Antibiofilm Properties. J. Pharm. Innov.

[39] Ajizah A (2018). Sensitivitas *Salmonella Typhimurium* Terhadap Ekstrak Daun *Psidium Guajava* L. Bioscientiae.

[40] Huang J, Zaynab M, Sharif Y, Khan J, Al-Yahyai R, Sadder M, Ali M, Alarab S. R, Li S (2024). Tannins as antimicrobial agents:Understanding toxic effects on pathogens. Toxicon.

[41] Budha Magar A, Shrestha D, Pakka S, &Sharma K. R (2023). Phytochemistry, biological, and toxicity study on aqueous and methanol extracts of Chromolaena odorata. TSWJ, 2023.

[42] Yusuf H, Husna F, Gani B. A, Garrido G (2021). The chemical composition of the ethanolic extract from Chromolaena odorata leaves correlates with the cytotoxicity exhibited against colorectal and breast cancer cell lines. J. Pharm. Pharmacogn. Res.

[43] Singh K. S, Anand S, Dholpuria S, Sharma J. K, Blankenfeldt W, Shouche Y (2021). Antimicrobial resistance dynamics and the One Health strategy:A review. Vet. World.

